# A Chinese family of autosomal recessive polycystic kidney disease identified by whole exome sequencing

**DOI:** 10.1097/MD.0000000000020413

**Published:** 2020-05-29

**Authors:** Jun Zhang, Li-meng Dai, Fu-rong Li, Bo Zhang, Jing-hong Zhao, Jin-bo Cheng

**Affiliations:** a Department of Nephrology, the key Laboratory for the Prevention and Treatment of Chronic Kidney Disease of Chongqing, Kidney Center of PLA, Xinqiao Hospital; b Department of Medical Genetics, Army Medical University (Third Military Medical University), Chongqing, China.

**Keywords:** autosomal recessive, compound heterozygous mutation, exome sequencing, polycystic kidney and hepatic disease 1, polycystic kidney

## Abstract

**Background:**

Autosomal recessive polycystic kidney disease (ARPKD) is an autosomal recessive hepatorenal fibrocystic syndrome. The majority of ARPKD patients progress to end-stage renal disease. Precise molecular diagnosis of ARPKD has proven valuable for understanding its mechanism and selecting optimal therapy.

**Methods:**

A Chinese family with ARPKD was recruited in current study. The clinical characteristics of ARPKD patient were collected from medical records and the potential responsible genes were studied by the whole exome sequencing (WES). Candidate pathogenic variants were validated by Sanger sequencing.

**Results:**

Both renal manifestation and hepatobiliary phenotype were observed. WES revealed compound heterozygous mutations of polycystic kidney and hepatic disease 1 genes, NM_138694: c.751G>T, (p.Asp251Tyr) and c.3998_4004delACCTGAA (p.Asn1333Thr fs × 13), which were confirmed by Sanger sequencing. Moreover, the mutations in the proband and its affected sib were co-segregated with the phenotype.

**Conclusions:**

The novel mutation in polycystic kidney and hepatic disease 1 gene identified by WES might be molecular pathogenic basis of this disorder.

## 1 Introduction

Autosomal recessive polycystic kidney disease (ARPKD, OMIM 263200) is an inherited disorder of congenital hepatorenal fibrocystic syndromes and is a cause of significant renal and liver-related morbidity and mortality in children.^[1,2]^ The incidence of ARPKD in the neonatal period is about 1/20 000, with the carrier frequency is approximately 1 in 70.^[3]^ The majority of ARPKD patients present in the neonatal period with enlarged echogenic kidneys. Renal disease is characterized by nephromegaly, hypertension, and varying degrees of renal dysfunction. The main causes of ARPKD are the mutations in the polycystic kidney and hepatic disease 1 (PKHD1) gene, which encode a membrane protein of 4074 amino acid (AA). More recently, DZIP1L was found to be a second gene involved in ARPKD pathogenesis.^[4]^

*PKHD1* is one of the largest human genes spanning a genomic segment of at least 470 kb, located on chromosome 6p21.1-p12.^[5]^ The longest transcript of *PKHD1* gene contains a reading frame with 67 exons, and codes the fibrocystin/polyductin protein with a short intracellular carboxyl terminal and a large extracellular amino terminal. The traditional method for ARPKD genetic testing is polymerase chain reaction (PCR)-based sequencing of the corresponding exons of *PKHD1* gene.^[6]^ By using this technology, numerous mutations of *PKHD1* gene have been identified in ARPKD patients. The majority of these mutations are missense, while frameshift, insertion, deletion, nonsense, and splice site mutations have been also found across the entire gene. With the progresses in next-generation sequencing, the extensive clinical utilization of genetic testing has been facilitated by rapid and relatively inexpensive sequencing technical innovation, such as whole exome sequencing (WES) and whole genome sequencing. For ARPKD patients, WES has been applied to identify the causative mutation.^[7,8]^ In this study, we reported novel mutations of *PKHD1* gene in a Chinese family with ARPKD that had never been described in a previous report.

## 2 Materials and methods

### 2.1 Subjects

The study was approved by the Ethics Committee of Xinqiao Hospital at Army Medical University (Chongqing, China). All participants provided written informed consent. The proband was a 29-year-old Chinese Han female, who was admitted to our Department of Nephrology at Xinqiao Hospital of Third Military Medical University (Chongqing, China) for evaluated serum creatinine. Her family including parents and a younger sister was recruited in current study. Blood samples were collected into graded negative pressure vacuum EDTA anticoagulant tubes.

### 2.2 Clinical evaluation

Clinical information was obtained from electronic health records. Abdominal ultrasound examination and CT scanning were performed for clinical diagnosis. Histopathology study of renal biopsy was applied to further understand its renal pathology. Renal ultrasonography in the parents disclosed normal findings and there was no family history of genetic renal diseases in either parent.

### 2.3 DNA extraction

Genomic DNA was isolated from the peripheral blood cells of these patients and their parents with a QIAamp DNA Blood MiniKit (Qiagen, Germany), according to the manufacturer's instructions.

### 2.4 Exome sequencing

WES of the DNA samples from the proband and the parents was performed by XX Co., Ltd. (Beijing, China). Briefly, genomic DNA samples were fragmented to 150∼200 bp by sonication and subjected to DNA library preparation using established Illumina paired-end protocol. Adaptor-ligated libraries were amplified via PCR. The samples were then sequenced on an Illumina Hiseq2500 platform (Illumina, San Diego, CA). All reads were aligned to the Human Reference genome (HG19) using the Burrows Wheeler Aligner (BWA) Multi-Vision software package. Single nucleotide variants and indels were identified using the SOAPsnp software and Samtools Indel Genotyper. The variants were annotated to Ensembl database using Sequence Variant Analyzer and were analyzed using the ATAV software. Two parallel reactions were done for each sample.

### 2.5 Bioinformatics analysis

To detect the potential pathogenic variants of the probands, bioinformatics analysis was performed based on online tools including PolyPhen2 and SIFT. Variants are classified according to the American College of Medical Genetics and Genomics standards and guidelines.^[9]^

### 2.6 Mutation validation

To confirm the mutation identified by WES, regular PCR was performed with primers spanning the mutation site. PCR products were directly sequenced using ABI 3730 Genetic Analyzer by using Sanger method.

## 3 Results

### 3.1 Clinical data

The evaluated serum creatinine of the proband was found during a routine physical examination. She had also suffered from thrombocytopenia for more than 8 year and discontinuously took some platelet-elevating drugs.

**Figure 1:**
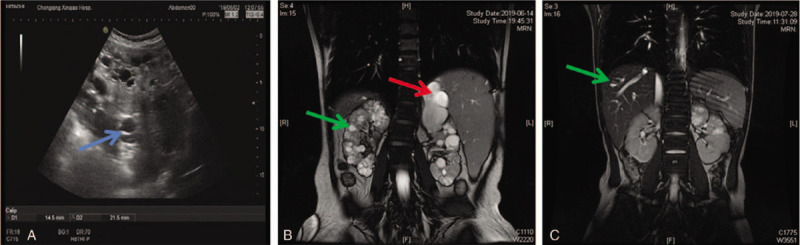
Clinical diagnostic images of proband. A: Abdominal ultrasound imaging showing the renal cysts of the proband (arrow). B: Magnetic resonance imaging showing renal cysts (green arrow), liver cysts and intrahepatic bile duct dilation of the proband (red arrow). C: Magnetic resonance imaging showing intrahepatic bile duct dilation of the affected sib (green arrow).

Laboratory test showed elevated levels of serum creatinine and uric acid, as well as impaired renal function (estimated glomerular filtration rate [egfr] 19.0 ml/min/1.73m^2^). Blood routine test showed decreased WBC, platelet and RBC, while urine routine tests showed mild proteinuria (0.33 g/d, 24-hour urine protein excretion) and suspected urinary occult blood (Table 1). The liver enzymes including ALT and AST were decreased. Ultrasound examination showed bilateral enlargement of the kidneys with hyperechogenicity and diffuse renal cysts of variable sizes (Fig. 1A). The diameter of the largest cysts on right kidney was 1.5 cm × 1.5 cm. Also, increased liver echogenicity, intrahepatic bile duct dilation and hypersplenotrophy were detected by ultrasound examination. In addition, CT scanning confirmed the bilateral enlargement of the kidneys with cysts and the intrahepatic bile duct dilation (Fig. 1B).

**Table 1:**
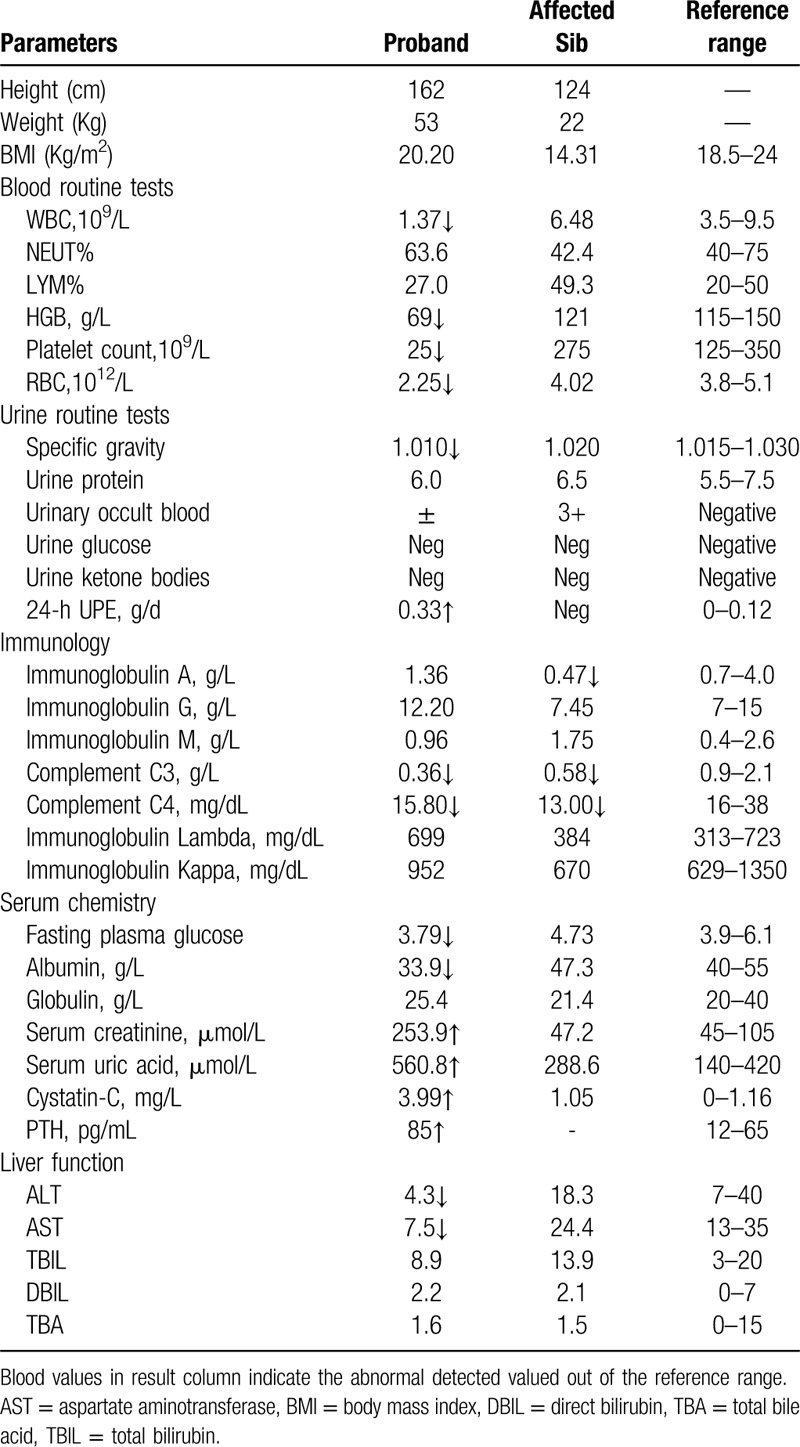
Laboratory data at presentation.

Considering the involvement of genetic factor, the younger sister of the proband, a 6-year-old girl, was screened by urine routine tests. Unfortunately, she had microalbumin (362 mg/L) and hematuresis, although her renal function (eGFR 150.0 ml/min/1.73m^2^) was normal. No other abnormality was detected in laboratory tests including blood routine test, immunology test and liver function test. In addition, no abnormality was detected by abdominal ultrasound examination except punctuated echogenicity in the kidneys. However, CT scanning showed mild intrahepatic bile duct dilation (Fig. 1C).

**Figure 2:**
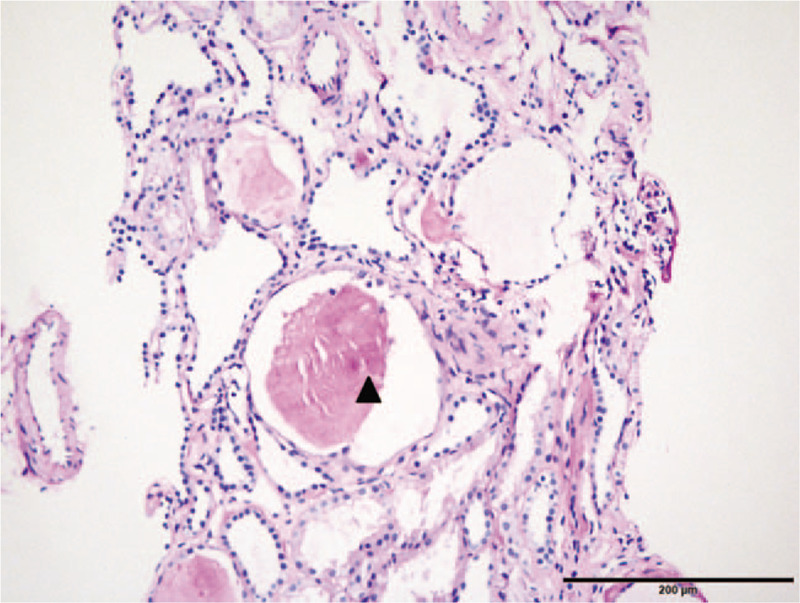
Histopathology study of renal biopsy from the affected sib. PAS staining showed the monolayer epithelium cells and the enlargement of renal tubules. Bar, 200 μm. PAS = periodic acid-schiff.

To further understand its renal pathology, histopathology study of renal biopsy from the affected sister was performed. Totally, 12 glomeruli were observed, and the inner sides of 3 glomeruli were covered with monolayer epithelium cells. Dilation and enlargement of collecting-duct and distal tubules in the kidney were detected (Fig. 2). All the immunological staining including IgA, IgG, IgM, complement C3, C4, C1q, K and λ was negative.

She orally took sirolimus (0.5 mg) per day. To ameliorate the anemia symptoms, Chinese patent drug (Shengplatelet capsule) was also used to improve platelet. However, the deterioration of renal function was hard to be reversed.

### 3.2 Exome sequencing and Sanger sequencing

**Figure 3:**
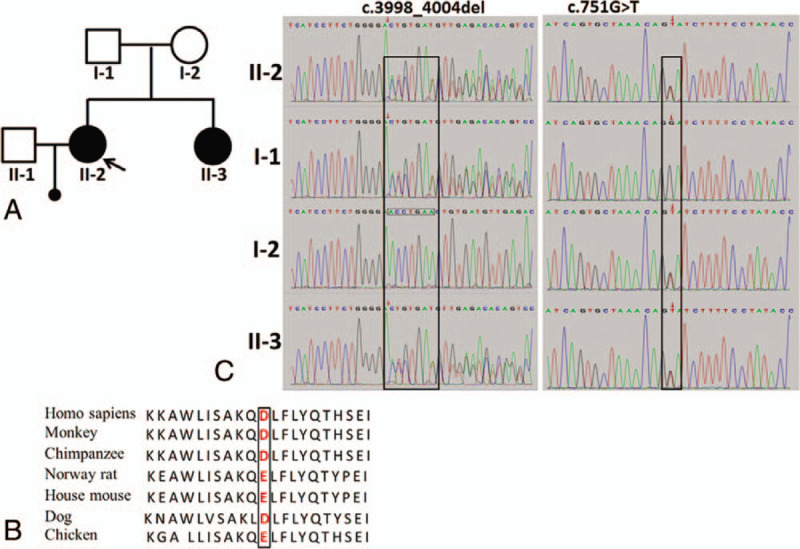
Identification of the novel mutation in the ARPKD family. A: Pedigree of the Chinese family. Affected family members are denoted in black. Arrow indicates the proband. B: Direct Sanger sequencing confirmed the compound heterozygous mutations of *PKHD1* gene. C: Evolutional conservation of p.Asp251Tyr among vertebrate species. PKHD1 = polycystic kidney and hepatic disease 1.

To further confirm its causative mutation of this family (Fig. 3A), genetic testing was performed based on WES. The isolated genomic DNA was fragmented to 150∼200 bp and subjected to DNA library and sequenced. Totally, the coverage was over 99.69% and the mean sequencing depth was about 107.7.

The sequencing analysis revealed that novel compound heterozygous mutations of *PKHD1* gene were found in the proband. One mutation was a missense NM_138694: c.751G>T, p.Asp251Tyr, and the other was a deletion, c.3998_4004delACCTGAA, p.Asn 1333Thr fs × 13. Sanger sequencing was performed to validate the identified variation (Fig. 3B). The 2 mutations were inherited from the parents. Moreover, the affected younger sister had the same genotype as that of the proband.

### 3.3 Mutation analysis

The longest transcript of *PKHD1* gene encodes a protein termed as fibrocystin, containing 4074 AA. The noval mutations of *PKHD1* gene were excluded from the single nucleotide polymorphism database (dbSNP), the ARPKD database and the Human Genetic Variation Database. The deletion variant (c.3998_4004del) led to reading frame shift, which resulted in a truncated protein. Due to the loss of the most functional domains of fibrocystin, this variant can be classified as “likely pathogenic” (PVS1 + PM2) according to the American College of Medical Genetics and Genomics standards and guidelines.^[9]^ The missense variant (c.751G>T) led to an AA change, located in the conserved AA sequences (Fig. 3C). The corresponding AA located near to the first immunoglobulin-like plexin-transcription-factor (IPT) domain. This missense variant was classified as “uncertain significance” (PM1 + PM2 + PP3).

## 4 Discussion

ARPKD is typically characterized by enlarged, echogenic kidneys with dilatation of the collecting ducts. ARPKD patients may (or may not) have liver disease consisting of dilated biliary ducts, congenital hepatic fibrosis, and portal hypertension. Most patients progress to end-stage renal disease at varying ages. The age of presentation is extremely variable. Based on the age of presentation and the severity of renal and liver disease, the variable clinical presentations of ARPKD are traditionally divided into different clinicopathologic groups: perinatal, neonatal, infantile, and juvenile.^[10]^ Although ARPKD is often suspected in late pregnancy or at birth and is characterized in severe neonatal forms, a minority of cases is less severe and symptoms do not develop until adolescence or adulthood.^[11]^

Our patient presented renal manifestations on age of 27. Evaluated serum creatinine was detected during routine examination, but no further abdominal ultrasound examination was performed at that time. She was grouped as CKD stage 4 for her eGFR was as low as 19.0 mL/min/1.73m^2^ on admission. As indicated by the pathological changes in her younger sister (6-year-old), it is rational to speculate that she might have pathological changes when she was a child; thus her disease progresses very slowly and the course of disease is very long. Besides renal manifestations, hepatobiliary pathological changes included intrahepatic bile duct dilation and hypersplenotrophy, all of which are typical presentations in ARPKD patients.

Ultrasonography is the diagnostic method of choice for assessing ARPKD patients because it is cost effective, painless, widely available, and does not require radiation or sedation. As cystic renal diseases are involved in many disorders such as autosomal dominant polycystic kidney disease (ADPKD),^[12]^ renal cysts and diabetes syndrome^[13]^ and glomerulocystic kidney disease,^[14]^ clinicians must carefully pay attention to the differential diagnosis, especially based on the renal ultrasonographic findings. Liver presentation such as congenital hepatic fibrosis is an indicative finding of ARPKD, but is rarely observed in other types of cystic renal diseases.

The molecular diagnosis of ARPKD is based on the finding of pathogenic variants in *PKHD1* gene identified by genetic testing. Although *DZIP1L* was found to be a second gene involved in ARPKD pathogenesis, few ARPKD patients were reported to be associated with this gene. The protein encoded by *PKHD1* gene is expressed in the human adult kidney, liver, and pancreas, and was shown to be localized to the primary cilia of inner medullary collecting duct cells.^[15]^

Traditionally, DHPLC and long-rang PCR sequencing were applied to detect *PKHD1* variants.^[16,17]^ To date, there are more than 700 variants deposited in the ARPKD database (Available at: http://www.humgen.rwth-aachen.de/index.php). Most *PKHD1* pathogenic variants are unique to single families.^[18]^ The relationship between genotype of *PKHD1* gene and phenotype of ARPKD has not been fully understood. In a study of 73 ARPKD patients, the pathogenic variant type did not correlate with kidney size or function.^[19]^ However, some studies showed that preliminary genotype-phenotype correlations could be drawn for the type of mutation rather than the site of the individual mutation. For example, patients with truncating mutations consistently have a severe renal presentation that results in neonatal death, while patients with at least 1 mutation typically have a milder renal presentation.^[20]^ Moreover, a study in 2003 showed that milder presentation of ARPKD required presence of AA substitution mutations.^[21]^ In addition, other genetic modifiers may also change phenotypes, even within the same family. The transcription factor HNF1-beta was reported to play a role in regulating *PKHD1* transcription.^[22]^ In our case, the patients had novel compound heterozygous mutations of “uncertain significance” and “likely pathogenic”, and the phenotype is mild. No variant in HNF1-beta was identified.

Besides the traditional PCR-based sequencing of the corresponding exons, more and more studies apply WES for ARPKD genetic analysis.^[23]^ One of the reasons is that this method is not as expensive as before. In addition, this method may help to identify mutations of different gene or genes that results in a similar clinical presentation. WES, as well as whole genome sequencing, will be extensively utilized in clinical genetic testing with the progresses made in next-generation sequencing in the future.

In summary, current study clearly showed the clinical diagnosis of ARPKD patient and confirmed its molecular basis of compound heterozygous mutations in *PKHD1* gene. The mutation in this case help to understand the relationship between genotype of *PKHD1* gene and phenotype of ARPKD, and WES is suitable for genetic diagnosis of inherited diseases like ARPKD.

## Acknowledgments

We wish to thank the patient and her family for participation in this study.

## Author contributions

ZJ, DLM and LFR performed the experiments. ZJ contributed the materials and reagent tools. ZJ and ZB analyzed the data. ZJH and CJB designed and guided this research study. ZB and CJB wrote the manuscript. All authors approved the final manuscript.

## References

[1] Bergmann C. Genetics of autosomal recessive polycystic kidney disease and its differential diagnoses. Front Pediatr 2018;5:221. 29479522 10.3389/fped.2017.00221 PMC5811498

[2] Fang Z Xu S Wang Y. Pathogenicity analysis of novel variations in Chinese Han patients with polycystic kidney disease. Gene 2017;626:433–41. 28578020 10.1016/j.gene.2017.05.046

[3] Zerres K Rudnik-Schöneborn S Steinkamm C. Autosomal recessive polycystic kidney disease. J Mol Med (Berl) 1998;76:303–9. 9587064 10.1007/s001090050221

[4] Lu H Galeano MCR Ott E. Mutations in DZIP1L, which encodes a ciliary-transition-zone protein, cause autosomal recessive polycystic kidney disease. Nat Genet 2017;49:1025–34. 28530676 10.1038/ng.3871 PMC5687889

[5] Onuchic LF Furu L Nagasawa Y. PKHD1, the polycystic kidney and hepatic disease 1 gene, encodes a novel large protein containing multiple immunoglobulin-like plexin-transcription-factor domains and parallel beta-helix 1 repeats. Am J Hum Genet 2002;70:1305–17. 11898128 10.1086/340448 PMC447605

[6] Zhang D Lu L Yang HB. Exome sequencing identifies compound heterozygous PKHD1 mutations as a cause of autosomal recessive polycystic kidney disease. Chin Med J (Engl) 2012;125:2482–6. 22882926

[7] Obeidova L Seeman T Elisakova V. Molecular genetic analysis of PKHD1 by next-generation sequencing in Czech families with autosomal recessive polycystic kidney disease. BMC Med Genet 2015;16:116. 26695994 10.1186/s12881-015-0261-3 PMC4689053

[8] Miyazaki J Ito M Nishizawa H. Intragenic duplication in the PKHD1 gene in autosomal recessive polycystic kidney disease. BMC Med Genet 2015;16:98. 26502924 10.1186/s12881-015-0245-3 PMC4623244

[9] Richards S Aziz N Bale S. Standards and guidelines for the interpretation of sequence variants: a joint consensus recommendation of the American College of Medical Genetics and Genomics and the Association for Molecular Pathology. Genet Med 2015;17:405–24. 25741868 10.1038/gim.2015.30 PMC4544753

[10] Blyth H Ockenden BG. Polycystic disease of kidney and liver presenting in childhood. J Med Genet 1971;8:257–84. 5097134 10.1136/jmg.8.3.257 PMC1469189

[11] Gunay-Aygun M Font-Montgomery E Lukose L. Correlation of kidney function, volume and imaging findings, and PKHD1 mutations in 73 patients with autosomal recessive polycystic kidney disease. Clin J Am Soc Nephrol 2010;5:972–84. 20413436 10.2215/CJN.07141009 PMC2879301

[12] Gimpel C Bergmann C Bockenhauer D. International consensus statement on the diagnosis and management of autosomal dominant polycystic kidney disease in children and young people. Nat Rev Nephrol 2019;15:713–26. 31118499 10.1038/s41581-019-0155-2 PMC7136168

[13] Verhave JC Bech AP Wetzels JF. Hepatocyte nuclear factor 1β-associated kidney disease: more than renal cysts and diabetes. J Am Soc Nephrol 2016;27:345–53. 26319241 10.1681/ASN.2015050544 PMC4731131

[14] Lennerz JK Spence DC Iskandar SS. Glomerulocystic kidney: one hundred-year perspective. Arch Pathol Lab Med 2010;134:583–605. 20367310 10.5858/134.4.583

[15] Menezes LF Cai Y Nagasawa Y. Polyductin, the PKHD1 gene product, comprises isoforms expressed in plasma membrane, primary cilium, and cytoplasm. Kidney Int 2004;66:1345–55. 15458427 10.1111/j.1523-1755.2004.00844.x

[16] Bergmann C1 Küpper F Schmitt CP. Multi-exon deletions of the PKHD1 gene cause autosomal recessive polycystic kidney disease (ARPKD). J Med Genet 2005;42:e63. 16199545 10.1136/jmg.2005.032318 PMC1735935

[17] Tong YQ Liu B Fu CH. Genetic analysis of the PKHD1 gene with long-rang PCR sequencing. J Huazhong Univ Sci Technolog Med Sci 2016;36:758–66. 27752906 10.1007/s11596-016-1658-8

[18] Bergmann C Senderek J Küpper F. PKHD1 mutations in autosomal recessive polycystic kidney disease (ARPKD). Hum Mutat 2004;23:453–63. 15108277 10.1002/humu.20029

[19] Gunay-Aygun M Tuchman M Font-Montgomery E. PKHD1 sequence variations in 78 children and adults with autosomal recessive polycystic kidney disease and congenital hepatic fibrosis. Mol Genet Metab 2010;99:160–73. 19914852 10.1016/j.ymgme.2009.10.010 PMC2818513

[20] Denamur E Delezoide AL Alberti C. Genotype-phenotype correlations in fetuses and neonates with autosomal recessive polycystic kidney disease. Kidney Int 2010;77:350–8. 19940839 10.1038/ki.2009.440

[21] Furu L Onuchic LF Gharavi A. Milder presentation of recessive polycystic kidney disease requires presence of amino acid substitution mutations. J Am Soc Nephrol 2003;14:2004–14. 12874454 10.1097/01.asn.0000078805.87038.05

[22] Hiesberger T Bai Y Shao X. Mutation of hepatocyte nuclear factor-1beta inhibits Pkhd1 gene expression and produces renal cysts in mice. J Clin Invest 2004;113:814–25. 15067314 10.1172/JCI20083 PMC362119

[23] Xu Y Xiao B Jiang WT. A novel mutation identified in PKHD1 by targeted exome sequencing: guiding prenatal diagnosis for an ARPKD family. Gene 2014;551:33–8. 25153916 10.1016/j.gene.2014.08.032

